# Frequent POLE-driven hypermutation in ovarian endometrioid cancer revealed by mutational signatures in RNA sequencing

**DOI:** 10.1186/s12920-021-01017-7

**Published:** 2021-06-22

**Authors:** Jaime I. Davila, Pritha Chanana, Vivekananda Sarangi, Zachary C. Fogarty, S. John Weroha, Ruifeng Guo, Ellen L. Goode, Yajue Huang, Chen Wang

**Affiliations:** 1 Department of Mathematics, Statistics and Computer Science, St Olaf College, Northfield, MN USA; 2 Department of Health Sciences Research, Mayo Clinic, Rochester, MN USA; 3 Division of Shared Resources, Fred Hutchinson Cancer Research Center, Seattle, WA USA; 4 Division of Medical Oncology, Mayo Clinic, Rochester, MN USA; 5 Division of Anatomic Pathology, Mayo Clinic, Rochester, MN USA

**Keywords:** Hypermutation, Mutational signatures, Ovarian endometrioid cancer, POLE, RNA-seq

## Abstract

**Background:**

DNA polymerase epsilon (POLE) is encoded by the POLE gene, and *POLE*-driven tumors are characterized by high mutational rates. *POLE*-driven tumors are relatively common in endometrial and colorectal cancer, and their presence is increasingly recognized in ovarian cancer (OC) of endometrioid type. *POLE*-driven cases possess an abundance of TCT > TAT and TCG > TTG somatic mutations characterized by mutational signature 10 from the Catalog of Somatic Mutations in Cancer (COSMIC). By quantifying the contribution of COSMIC mutational signature 10 in RNA sequencing (RNA-seq) we set out to identify *POLE*-driven tumors in a set of unselected Mayo Clinic OC.

**Methods:**

Mutational profiles were calculated using expressed single-nucleotide variants (eSNV) in the Mayo Clinic OC tumors (n = 195), The Cancer Genome Atlas (TCGA) OC tumors (n = 419), and the Genotype-Tissue Expression (GTEx) normal ovarian tissues (n = 84). Non-negative Matrix Factorization (NMF) of the mutational profiles inferred the contribution per sample of four distinct mutational signatures, one of which corresponds to COSMIC mutational signature 10.

**Results:**

In the Mayo Clinic OC cohort we identified six tumors with a predicted contribution from COSMIC mutational signature 10 of over five mutations per megabase. These six cases harbored known POLE hotspot mutations (*P286R*, *S297F*, *V411L*, and *A456P*) and were of endometrioid histotype (*P* = 5e−04). These six tumors had an early onset (average age of patients at onset, 48.33 years) when compared to non-POLE endometrioid OC cohort (average age at onset, 60.13 years; *P* = .008). Samples from TCGA and GTEx had a low COSMIC signature 10 contribution (median 0.16 mutations per megabase; maximum 1.78 mutations per megabase) and carried no POLE hotspot mutations.

**Conclusions:**

From the largest cohort of RNA-seq from endometrioid OC to date (n = 53), we identified six hypermutated samples likely driven by POLE (frequency, 11%). Our result suggests the clinical need to screen for POLE driver mutations in endometrioid OC, which can guide enrollment in immunotherapy clinical trials.

**Supplementary Information:**

The online version contains supplementary material available at 10.1186/s12920-021-01017-7.

## Background

DNA polymerase epsilon (POLE) is a key member of the DNA polymerase family and is involved in error correction during replication [1]. Somatic mutations in the exonuclease domain of *POLE* have been identified in 7–12% of uterine endometrial cancers, in 1–2% of colorectal cancers, and at low frequencies in stomach cancer, glioblastoma, breast cancer, and others [2–5]. Recurrent hotspot mutations include *P286R*, *S297F*, and *V411L*, which are characterized by ultramutation [5, 6]. Patients with *POLE*-driven endometrial cancer have a favorable prognosis, a higher neoantigen load, an increased number of tumor-infiltrating lymphocytes, and they may benefit from immunotherapy [6–8].

Somatic *POLE* mutations are uncommon in serous ovarian cancer (OC) [9, 10] but the presence of *POLE* mutations is increasingly recognized in ovarian endometrioid cancer (OEC) [10–14]. Patients with *POLE*-driven OEC have earlier disease onset, and an increased number of *CD8* + intraepithelial tumor-infiltrating lymphocytes [11, 14]. The prevalence of *POLE-*driven tumors in different OEC studies ranges from 3 to 13% [10–12, 14–16], and is likely driven by sample size, cohort selection criteria, and type of mutation detection assay. Interestingly, in a Japanese cohort of concurrent ovarian and endometrial cancer, the frequency of *POLE* mutations was high (five of 8 cases; 62%) detected by Sanger sequencing [13]; however, the reported *POLE* mutations (*Q292E*, *E396V*, *D287N*, and *N293D*) do not correspond to known hotspot mutations. This result highlights the need to ascertain the effects of particular *POLE* mutations and whether they are accompanied by hypermutation.

*POLE*-driven tumors are associated with a distinct mutational signature found in whole-genome sequencing from tumor-normal pairs which is characterized by a high number of TCT > TAT and TCG > TTG mutations [4, 17]. Such distinct mutational profile is known as COSMIC mutational signature 10 [17]. Exome sequencing detected an abundance of COSMIC mutational signature 10 in OEC cases with known *POLE* hotspot mutations [16]. Previously, we developed a novel method for inferring and quantifying distinct mutational signatures using RNA sequencing (RNA-seq) data [18, 19]. We applied this method in tumor-only fresh-frozen RNA sequencing (RNA-seq) samples in endometrial cancer and colorectal cancer tumors from The Cancer Genome Atlas (TCGA) and identified *POLE* cases with high specificity and sensitivity [19].

Using previously published RNA-seq from a Mayo Clinic OC cohort (n = 195), which included the largest set of OEC transcriptomes to date (n = 53), we sought to leverage the mutational signatures approach to identify *POLE*-driven cases and to characterize their clinical characteristics [20, 21].

## Methods

### Mayo Clinic and public data selection and RNA-seq

Selected participants were patients who were at least 20 years old and were ascertained at Mayo Clinic from 1992 through 2009 within one year after receiving a pathologically confirmed diagnosis of primary invasive epithelial OC, fallopian tube cancer, or primary peritoneal cancer. (Table 1). Patients were treated using standard first line platinum-based chemotherapy. Tumors were snap frozen immediately after surgery and stored at − 80 °C. A gynecologic pathologist confirmed the clinical diagnoses and verified the tumor histology and grade and the presence of 70% tumor content before RNA extraction from fresh frozen tissue. As described previously [20, 21] transcriptomic sequencing was performed in four batches with TruSeq Library Preparation kits (Stranded Total RNA Library Preparation Kit or RNA Library Preparation Kit v2; Illumina, Inc) and sequenced on the Illumina HiSeq 2000 sequencer with 100–base pair paired-end reads. All patients gave informed consent; all protocols were approved by the Mayo Clinic Institutional Review Board.

**Table 1:** Characteristics of the 195 patients in the Mayo Clinic ovarian cancer cohort

Feature	Value
Age at diagnosis, mean (SD), y	62 (12)
Histology, no. (%)	
Serous	114 (58)
Endometrioid	53 (27)
Clear cell	14 (7)
Undifferentiated	8 (4)
Mucinous	3 (2)
Other	3 (2)
Grade, no. (%)	
1	20 (10)
2	24 (12)
3	146 (75)
Unknown	5 (3)
Stage, no. (%)	
I	45 (23)
II	15 (8)
III	100 (51)
IV	33 (17)
Unknown	2 (1)

We also used OC RNA-seq from TCGA (n = 419) and from normal ovarian tissue from the Genotype-Tissue Expression (GTEx) project (Broad Institute) (n = 84) [9, 22].

### Bioinformatics methods

RNA-seq of Mayo Clinic, TCGA, and GTEx data sequencing reads were processed through the Mayo Clinic MAP-RSeq v.2.1.5 computational workflow, and variants were calculated with RVboost 0.1 [18, 23]. We considered expressed single nucleotide variants from RVboost with a Q score greater than 10%, read depth greater than 10, a minor allelic frequency less than 2% in the 1000 Genomes Project [24], and not present in recurrent expressed single-nucleotide variants identified in RNA-seq from adjacent normal tissue. The RNA-seq capture region was defined as positions with 20 × coverage as calculated by the Genome Analysis Toolkit (GATK; Broad Institute). Samples with read depth over 20 × at less than five million positions were excluded from this analysis. Tumor mutation burden (TMB) was calculated as the number of considered expressed variants per capture region × 10^6^.

We used the mutational signatures v2 from the Catalogue of Somatic Mutations in Cancer (COSMIC) [25] where the mutational profiles are represented as the proportion of each substitution type (C > A, C > G, C > T, T > A, T > C, and T > G) and its trinucleotide context (the nucleotide before and after each mutated base) [17]. For the detection of mutational signatures and their contribution to each sample, we used R version 3.4.2 (R Foundation) with the MutationalPatterns v1.4.3 package [26]. To measure the similarity between two mutational signatures we used the cosine similarity as implemented in the function *cos_sim* in the MutationalPatterns v1.4.3 package. The cosine similarity takes values between 0 and 1, with a value close to 1 if there is great similarity between signatures and close to 0 if the two signatures are dissimilar.

### Statistical analysis

We used R version 3.4.2 with the tidyverse 1.2.1 package to perform statistical analyses and generate graphs. Whiskers in the boxplots correspond 75th (and 25th) Quantile ± 1.5 IQR (Interquantile range) and outliers correspond to values outside this range. Two-sided Mann–Whitney tests (Wilcoxon rank sum tests) were used for comparisons of contributions of *POLE* mutational signature across the Mayo OC cohort and TCGA/GTEx, and age across patients with and without *POLE* mutations. Histology findings from patients with and without *POLE* mutations were compared with the Fisher exact test.

## Results

The Mayo Clinic OC cohort with existing RNA-seq consisted of 195 patients whose clinical characteristics are described in Table 1 [20, 21]. This cohort, contains an abundance of nonserous histologies (81 of 195) and includes 53 with OEC, which makes it the largest OEC RNAseq collection to date.

**Fig. 1:**
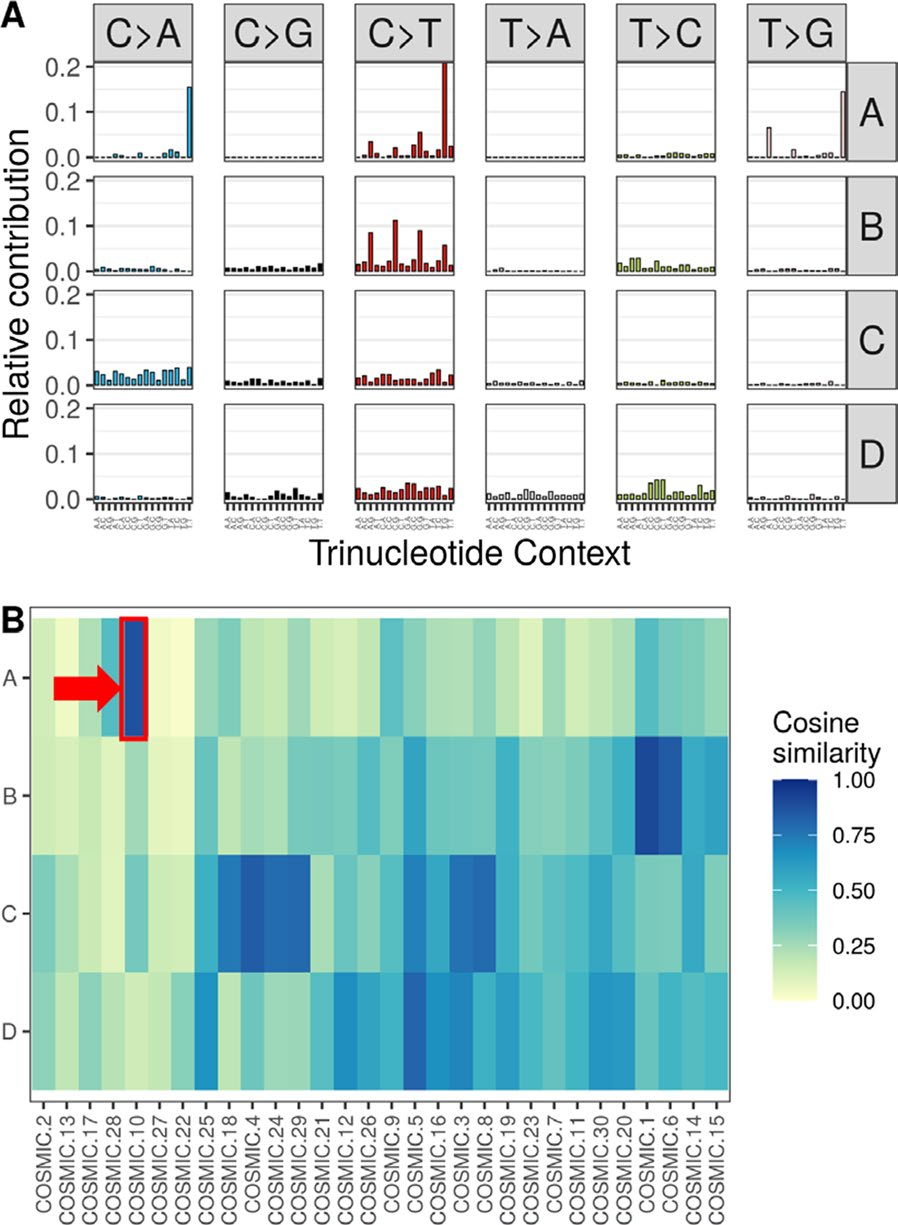
Mutational Signatures from RNA Sequencing of Tissues with and without Ovarian Cancer (OC). **a** The four mutational signatures according to trinucleotide context in the Mayo Clinic and TCGA OC tumors combined. **b** Heatmap of the cosine similarity of the four mutational signatures compared against the reference Catalogue of Somatic Mutations in Cancer (COSMIC) signatures. COSMIC signatures are grouped on the x-axis by using hierarchical clustering using the average method according to their cosine similarities. Mutational signature A has a high cosine similarity against COSMIC mutational signature 10 and it is denoted in red

Mutational profiles from eSNV were calculated in the Mayo Clinic OC tumors (n = 195), TCGA OC tumors (n = 419), and the GTEx normal ovarian tissues (n = 84). Using Non-negative Matrix Factorization (NMF) [26] we were able to approximate each sample’s mutational profile as a combination of four distinct mutational signatures (Fig. 1a and Additional file 1: Fig. S1). Using the cosine similarity, we established a high degree of resemblance between the approximated mutational profiles from NMF and the original mutational profile (median cosine similarity, 0.92; interquartile range, 0.89–0.93) (Additional file 1: Fig. S2).

Using the cosine similarity, the four mutational signatures were compared against the COSMIC mutational signatures catalog v2 (Fig. 1b) [17]. Signature B has a cosine similarity of 0.91 against COSMIC signature 1 which is associated with aging [27]. Signature C has cosine similarities of 0.85 and 0.77 against COSMIC signatures 4 and 3, which are associated with tobacco exposure and defective double strand repair respectively [17]. Signature D has a cosine similarity of 0.81 against COSMIC signature 5, a signature found in all cancer types [17].

Of note, signature A has a cosine similarity of 0.88 to COSMIC signature 10, which is associated with *POLE* defects [2]. Six samples in the Mayo Cohort have a similar mutational distribution characterized by an enrichment of the *POLE* contribution (Additional file 1: Fig. S1B and C).

**Fig. 2:**
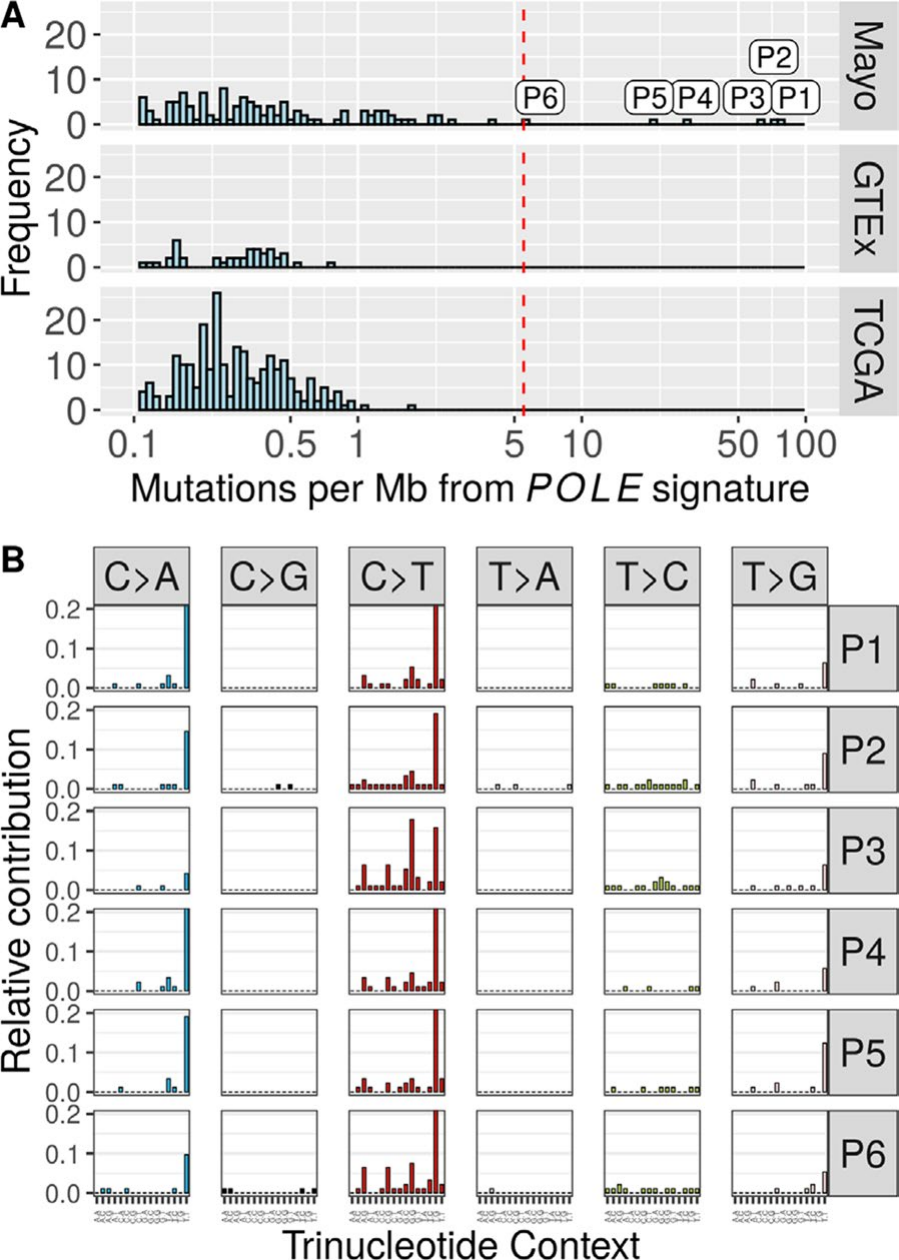
Identification of DNA Polymerase Epsilon (*POLE*)-Driven Ovarian Cancer Samples. **a** Distribution of the tumor mutation burden (TMB) attributed to the *POLE* signature across the cohorts from Mayo Clinic (Mayo), Genotype-Tissue Expression (GTEx), and The Cancer Genome Atlas (TCGA), which allows the identification of six samples (P1–P6) with high *POLE* contribution and more than five mutations per megabase. The red dashed line indicates a threshold used to separate between *POLE* positive and negative cases. **b** Mutational profiles of six POLE-driven Mayo Clinic OEC tumors according to the frequency of trinucleotide context for the six samples; their distinct C > A and C > T peaks are associated with the *POLE* signature. Red line denotes the threshold used for the outlier direction (5.7 Mut per Mb)

The *POLE* signature contribution of such six samples (of 195) from our Mayo OC were deemed as outliers (> 5.74 Mut per megabase) (see Methods and Additional file 1: Fig. S3). Normal samples from GTEx and serous OC samples from TCGA had a median of 0.16 mutations per megabase; maximum 1.78 mutations per megabase of the *POLE* signature and can be used as negative *POLE* controls (Fig. 2a). The six outlier Mayo OC samples have a higher *POLE* signature contribution (median 46.34 mutations per megabase; *P* = 2.37E−05) when compared to the negative *POLE* controls (Fig. 2a). The individual mutational profiles for each of those six samples are shown in Fig. 2b.

Within these six samples, we found expressed mutations corresponding to the *POLE* hotspot COSMIC mutations *P286R*, *S297F*, *V411L*, and *A456P* (Table 2 and on Additional file 1: Fig. S3) [28]. No other samples in the Mayo OC, the TCGA OC, or the normal ovarian GTEx cohort harbored expressed mutations in any of the *POLE* hotspots.

**Table 2:** Clinical Characteristics of six *POLE* Samples from the Mayo Clinic Ovarian Cancer Cohort

Sample	*POLE* mutation	*POLE* TMB	Age, years	Histology	Stage	Grade	Vital status
P1	*A456P*	75.2	46	Endometrioid	III	2	Alive
P2	*S297F*	71.7	49	Endometrioid	I	3	Alive
P3	*V411L*	64.1	49	Endometrioid	III	3	Deceased
P4	*P286R*	28.6	46	Endometrioid	I	2	Alive
P5	*P286R*	21.6	49	Endometrioid	I	1	Alive
P6	*V411L*	5.8	51	Endometrioid	I	1	Alive

*POLE* DNA polymerase epsilon, *TMB* tumor mutation burden

All six samples were of endometrioid histotype (*P* = 5e−04) and constituted 11% of OEC cases. Average age at onset was earlier (48.33 years) than in the non-*POLE* OEC cohort (60.13 years; *P* = 0.008). Four of these six samples corresponded to stage 1, and two corresponded to stage 3 (Table 2).

## Discussion

RNA-seq is routinely used for transcriptome quantification and fusion detection, as illustrated by our previous studies where we characterized gene expression and fusions across different OC histology types [20, 21]. This study constitutes a novel reuse of this OC RNA-seq cohort, along with multiple public RNA-seq datasets including TCGA and GTEx, to enable clinically significant discoveries of previously unidentified *POLE* altered cases. Furthermore, our bioinformatics approach can identify the distinct *POLE* mutational signatures in RNA-seq as well as confirm the expression of *POLE* hotspot mutations. This adds to the clinical utility of RNA-seq which can already detect fusions and calculate tumor mutational burden in a single clinical assay, as opposed to DNA-seq [29, 30].

POLE-driven tumors have a favorable prognosis, an increased number of tumor-infiltrating lymphocytes, and can benefit from immunotherapy. Despite these distinct characteristics of *POLE*-driven tumors, *POLE* mutation status is not routinely evaluated in a clinical setting for OEC. By using RNA-seq on the largest OEC cohort to our knowledge (n = 53), we found six of 53 OEC samples (11%) that were *POLE*-driven. Those six samples had a highly specific mutational profile corresponding to the well-characterized *POLE* COSMIC signature 10 profile, and *POLE* hotspot mutations (*P286R*, *S297F*, *V411L*, and *A456P*). Two of the 6 patients in the group of *POLE* cases had advanced-stage OEC with relatively high recurrence risks; therefore, OEC patients with *POLE*-driven tumors can be eligible for immunotherapy trials if their cancers progress or recur.

The tumor mutational burden (TMB) attributed to COSMIC signature 10 of our *POLE* cases spans from over five to more than 75 mutations per megabase. Patients with higher TMB attributed to COSMIC signature 10 have worse clinical characteristics and outcomes than among non-POLE OECs; the sample with the lowest TMB was from a patient with a low-grade, stage 1 cancer, and the three samples with the highest TMB correspond to higher-grade or higher stage cancers. However, the limited number of *POLE* cases in this study precluded any statistical analysis, and further independent studies with larger sample sizes are necessary to validate and confirm such a trend.

The smaller number of mutations in RNA-seq as compared to Whole Genomic Sequencing (WGS) reduces the statistical power to find known signatures present at lower levels and to distinguish the contribution of closely related and flat signatures. To illustrate this point, COSMIC signature 3 which is associated with defective homologous recombination and is prevalent in OC had only cosine similarity of 0.77 against signature C, while signature 4 which is associated with tobacco and not known to play a role in OC had a cosine similarity of 0.84 against signature C. Future studies to quantify the limit of detection of mutational signatures in RNA-seq are warranted.

## Conclusions

Using RNA-seq mutational signatures from the largest OEC cohort to date (n = 53), we found that 6 hypermutated samples (11%) had evidence of *POLE*-driven tumors.

## Supplementary Information

**Additional file 1.** Supplementary Figures.

## Data Availability

The datasets and code generated during and/or analyzed during the current study are available in https://github.com/jdavilal/mutational_sig_oec.

## Abbreviations

COSMIC: Catalogue of Somatic Mutations in Cancer
GTEx: Genotype-Tissue Expression
MMR: mismatch repair
MSK-IMPACT: Memorial Sloan Kettering integrated mutation profiling of actionable cancer targets
OC: ovarian cancer
OEC: ovarian endometrioid cancer
POLE: DNA polymerase epsilon
RNA-seq: RNA sequencing
TCGA: The Cancer Genome Atlas
TMB: tumor mutation burden
