# Supplementary material for: Frequent POLE-driven hypermutation in ovarian endometrioid cancer revealed by mutational signatures in RNA sequencing
Source: BMC Med Genomics. 2021 Jun 22;14:165. doi: 10.1186/s12920-021-01017-7 (PMC8218518; doi:10.1186/s12920-021-01017-7)
Supplement: Supplementary file 1 — Additional file 1. Supplementary Figures. [file 12920_2021_1017_MOESM1_ESM.docx]

**Additional file 1**

Frequent POLE-Driven Hypermutation in Ovarian Endometrioid Cancer Revealed by Mutational Signatures in RNA Sequencing

Jaime I. Davila, PhD^* 1,2^

Pritha Chanana, MS^3^

Vivekananda Sarangi, MS^2^

Zachary C. Fogarty, MS^2^

S. John Weroha, MD, PhD^4^

Ruifeng Guo, MD, PhD^5^

Ellen L. Goode, PhD^2^

Yajue Huang, MD, PhD^5^

Chen Wang, PhD^2^

**Author Affiliations**:

* Corrresponding author. Correspondence to [davila3@stolaf.edu](mailto:davila3@stolaf.edu)

1. Department of Mathematics, Statistics and Computer Science St Olaf College, Northfield, Minnesota
2. Department of Health Sciences Research, Mayo Clinic, Rochester, Minnesota
3. Division of Shared Resources, Fred Hutchinson Cancer Research Center, Seattle, Washington.
4. Division of Medical Oncology, Mayo Clinic, Rochester, Minnesota
5. Division of Anatomic Pathology, Mayo Clinic, Rochester, Minnesota

**Supplementary figures**

**Supplementary figure 1. Signature contribution across samples.** A). Absolute signature contribution in Mutations per megabase for *POLE* (red), B (green), C (cyan) and D (purple) across the Mayo, GTEx and TCGA measured in mutations per Mb. B) Percent signature contribution across all samples. Samples were clustered according to the similarity of their mutational profiles. C) Percent signature contribution for cluster of 6 samples with high *POLE* contribution.


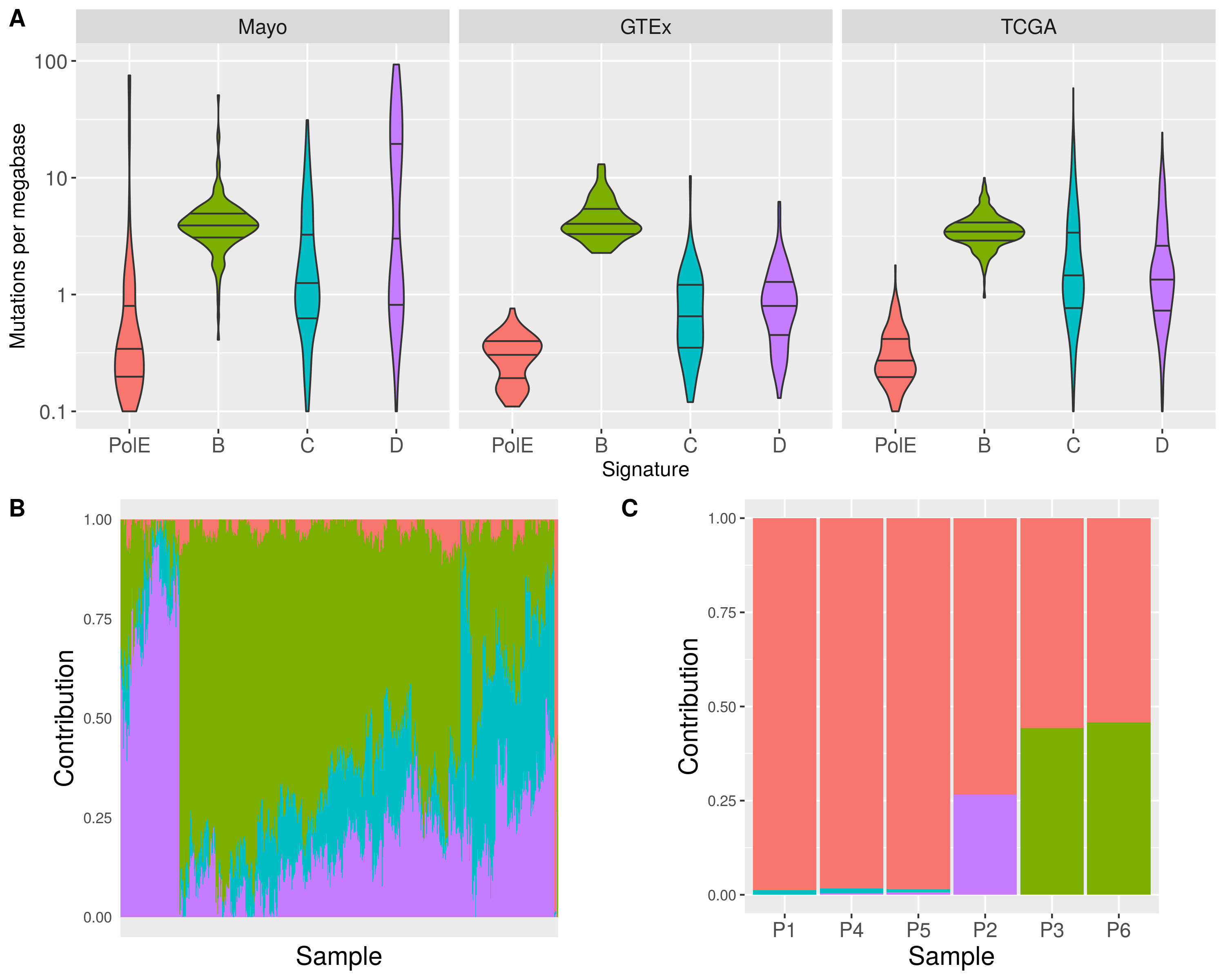


**Supplementary figure 2. Cosine similarity distribution between original and reconstructed mutational profile.** Histogram of cosine similarity between the original mutational signature and the reconstructed combination of the four mutational signatures identified in the Non-Negative Matrix factorization analysis.


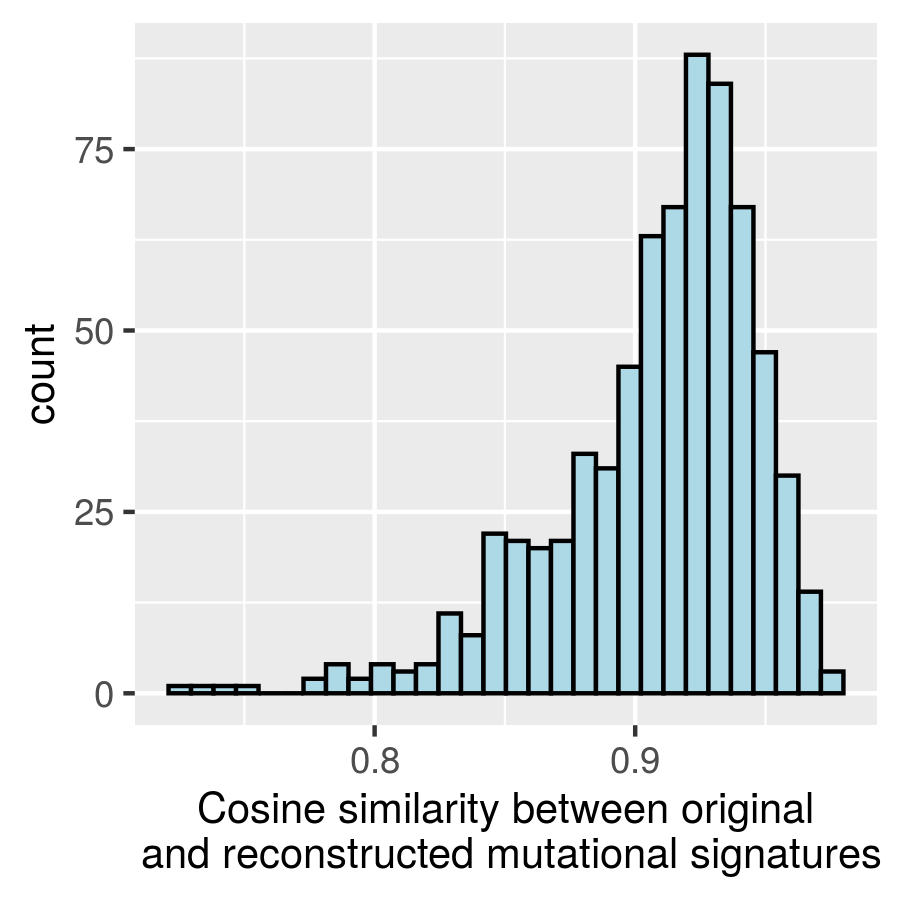


**Supplementary figure 3. Distribution of mutations per megabase from POLE signature for the Mayo Cohort.** Boxplot of mutations per Mb from POLE signature. Outlier samples are labeled in the graph. Red dotted line represents the outlier threshold.


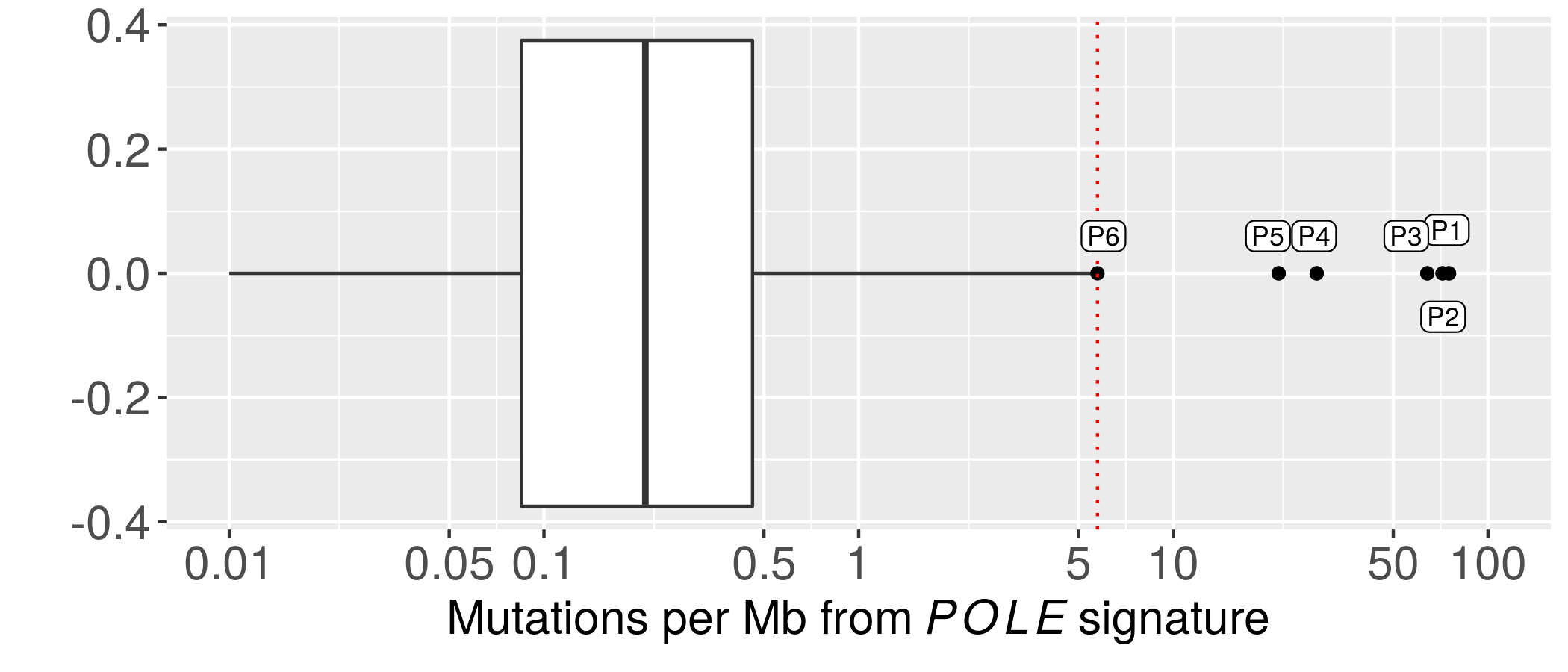


**Supplementary figure 4. Histogram of recurring COSMIC POLE hotspots.** Lollipop plot depicting the recurring mutations in POLE as found in the COSMIC database, as well as their frequency in this database. Only mutations occurring in over 10 samples were considered.

**
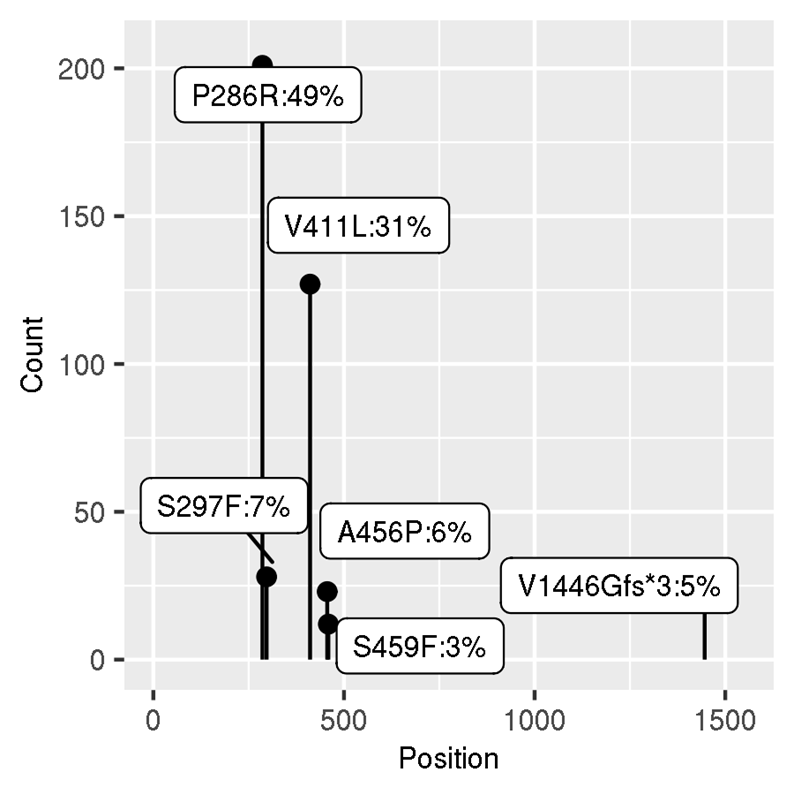
**
